# A Novel Striated Membrane Contact Site in Vestibular Hair Cells

**DOI:** 10.1111/boc.70082

**Published:** 2026-09-29

**Authors:** Nicolas Brouilly, Nathalie Pujol, Fabrice Richard, Chantal Cazevieille, Jennifer Stone, Ruth Anne Eatock, Rémy Pujol

**Affiliations:** ^1^ IBDM, Institut de Biologie du Développement de Marseille Aix Marseille Univ Marseille France; ^2^ CIML, Aix Marseille Univ, INSERM, CNRS Turing Centre for Living Systems Marseille France; ^3^ INM, Institut des Neurosciences de Montpellier INSERM‐Université de Montpellier Montpellier France; ^4^ Department of Otolaryngology/Head and Neck Surgery, and the Virginia Merrill Bloedel Hearing Research Center University of Washington Seattle Washington USA; ^5^ Department of Neurobiology University of Chicago Chicago Illinois USA

## Abstract

The mammalian vestibular system has two types of sensory receptor hair cells (HCs), each with different neurotransmission mechanisms. Type II HCs use ribbon synapses to transmit neurotransmitters like glutamate to bouton terminals of afferent neurons. Type I HCs are nearly engulfed by a calyceal afferent ending. They regulate afferent activity through both quantal and non‐quantal transmission (NQT), the latter faster than classic neurotransmitter release and potentially key to stabilizing vision and balance during rapid head movements. Here, we describe a novel striated contact, present only in the mouse type I HC and its calyceal afferent ending. Electron tomography reveals periodic striations on both the HC and calyx plasma membranes. These contact sites are intimately associated with a plasma membrane mitochondria contact site on the type I HC side of the synapse. Given that mitochondrion–plasma membrane contact sites in other systems regulate local ion homeostasis, this distinctive arrangement has the potential to serve or modulate NQT at the type I HC–calyx synapse.

## Graphical abstract

The mammalian vestibular system features type I and II hair cells (HCs) with distinct neurotransmission: type I uses both ribbon synapses and rapid non‐quantal transmission (NQT). We reveal a novel striated membrane contact between type I HCs and calyceal afferents, linked to atypical mitochondria. This structure may modulate NQT, offering insights into fast synaptic signaling for balance and vision stability.

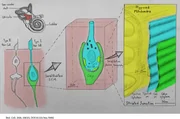

## 1 Main Text

Balance, locomotion, and steady gaze depend on multiple sensory inputs, with the vestibular inner ear directly supplying the fastest signals that allow stability during motion transients. In the vestibule of amniotes, there are two types of sensory receptor cells, called hair cells (HCs), which differ dramatically in many respects, including their mode of neurotransmission to primary afferent neurons (reviewed in (Eatock and Songer 2011)). The afferent synapses of type II HCs are ribbon synapses, with glutamate vesicles arrayed around a presynaptic ‘ribbon’ and facing a postsynaptic afferent bouton. In contrast, type I HCs have a unique feature in that they are nearly fully engulfed by a calyceal afferent ending (Figure 1A,B). Type I HCs form ribbon synapses with the calyx (Lysakowski 1999; Michanski et al. 2023). In immature rodents, the type I ribbon synapses release glutamate vesicles onto calyces (Sadeghi et al. 2014; Songer and Eatock 2013) but over development, the incidence of release decreases substantially in type I HCs relative to type II HCs (Spaiardi et al. 2022). Over the same period, type I HCs develop a unique non‐quantal transmission (NQT). This NQT has a component that is faster than classic quantal transmission (Songer and Eatock 2013) and may help explain the remarkable ability of the vestibular system to stabilize vision and balance during rapid head motions (Govindaraju et al. 2023).

**Figure 1:**
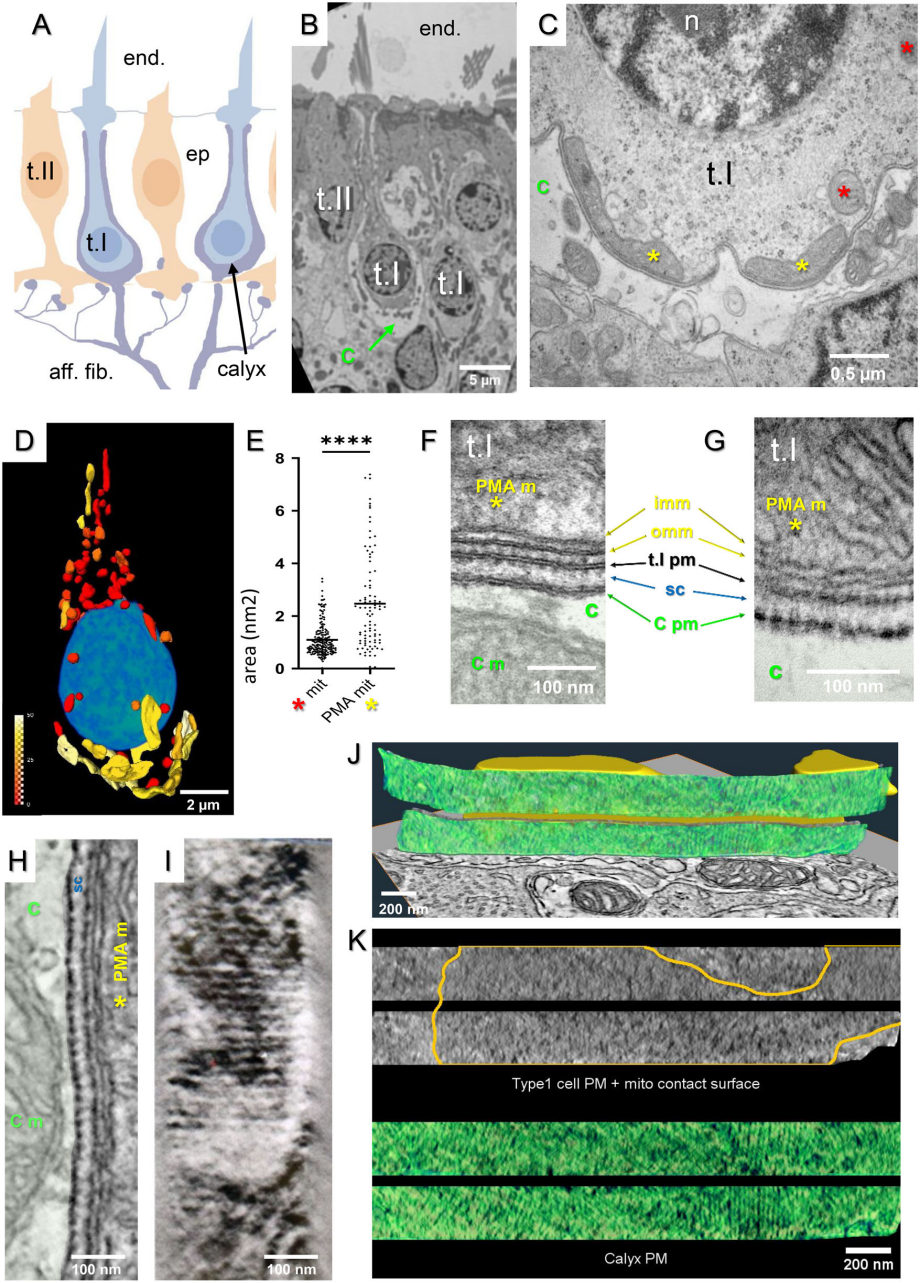
A novel striated junction between the type I hair cell and its calyceal afferent ending in the mouse utricle. (A) Schematic drawing of the utricular epithelium organization in an adult mouse (t.I, type I hair cell; t.II, type II hair cell; c, calyx; end, endolymph; ep., sensory epithelium; aff. fib., afferent fibers). (B) Electron micrograph of mouse utricular epithelium showing t.I, t.II, and calyx (c). (C) Adult t.I transverse section located right below the nucleus; yellow *, PMA mitochondria; red *, other mitochondria. (D) Segmentation from serial block‐face (SBF) imaging on t.I showing all mitochondria. Blue ovoid: t.I nucleus. The proportion of contact with the plasma membrane for each mitochondrion is represented in color ranging from no contact (red) to 50% of surface contact (white–yellow); (see Supporting Video S1). (E) Area (nm^2^) of t.1 mitochondria depending on their proximity to the plasma membrane. We binned the mitochondria in 2 groups with either < 20% (*n* = 232) or > 20% (*n* = 93) surface contact on 3 combined segmented SBF cells, unpaired *t*‐test **** *p*>0,0001. (F, G) High magnification electron micrographs of striations. Depending on the angle of the section, striations can be visible or not, highlighting the need for electron tomography (imm: inner mitochondrial membrane; omm: outer mitochondrial membrane; m: mitochondria; pm: plasma membrane; t.I: type I HC; c.: calyx; sc: synaptic cleft; calyx is pseudo‐colored in semi‐transparent green). (H‐K): ssET tomogram. (H, I) transversal image (H) and corresponding *en face* view (I) of a tomogram highlighting the periodic striations on the calyx membrane (sc: synaptic cleft; calyx is pseudo‐colored in semi‐transparent green). (J) 3D rendering of serial tomograms segmentation of the calyx membrane with an *en face* view (green) and the PMA mitochondria (yellow) (see Supporting Video S2). (K) Pseudo‐colored plasma membranes from segmentations in two serial tomograms (grey: type I HC plasma membrane, green: calyx plasma membrane, orange contour: the surface of contact between the mitochondria and the type I cell plasma membrane).

In the type I HC, as in other cochlear or vestibular sensory HCs, mitochondria are distributed throughout the whole cytoplasm but also form two distinct clusters with likely different physiological functions. One cluster sits just below the cuticular plate, a large apical structure into which the mechanosensory stereocilia implant, and is therefore well‐positioned to support mechanoelectrical transduction (Vranceanu et al. 2012). Another cluster is found at the basal synaptic pole, well‐positioned to sustain neurotransmission (Lysakowski et al. 2022). With transmission electron microscopy (TEM) on ultrathin sections of this basal synaptic cluster, we found that several mitochondria closely abut the plasma membrane (Figure 1C; adult mouse utricle). These plasma membrane‐apposed (PMA) mitochondria were detected specifically in type I HCs, throughout the utricular epithelium, in both physiologically and morphologically distinct central (‘striolar’) and peripheral (‘extrastriolar’) zones, and were also noted in the posterior crista ampullaris (Figure S1). To our knowledge, PMA mitochondria have not been described in type I HCs or other HC types in any species.

Serial block‐face (SBF) scanning electron microscopy of whole type I HCs in mouse utricles revealed the localization, size, and proportions of the PMA mitochondria (Figure 1D). We confirmed that these mitochondria dominated the basal half of the cell below the cell nucleus, and that they contacted 10%–20% of the type I HC plasma membrane at this level. Relative to other mitochondria, PMA mitochondria were larger and more elongated, spreading along the plasma membrane (Figure 1D,E and Figure S4; *n* = 325 mitochondria in 3 different SBF‐segmented type I HCs). Using TEM, we observed a remarkable alignment of 4 sets of membranes: the plasma membranes of the calyx and the type I HC, together with the inner and outer membranes of the PMA mitochondrion in the HC (Figure 1F,H). These observations revealed two striking features: (1) the outer membrane of each PMA mitochondrion is very closely apposed to the type I HC plasma membrane, with only a 10–15 nm gap between the two structures (Figure 1F,H); and (2) alternating light and dark electron‐dense material is visible on the plasma membranes of both the type I HC and the postsynaptic calyx (Figure 1G). We refer to these specialized zones as “striated contacts”. Because the visibility of membrane striations in single thin sections depends on the plane of sectioning, only electron tomography can unambiguously resolve them. Using serial section electron tomography (ssET), we confirmed the presence of striations on the plasma membranes of both the type I HC and the facing calyx (Figure 1G,H). In our tomograms (*n* = 5, in 5 different type I HCs), every PMA mitochondrion was apposed to a striated membrane domain. Conversely, we did not observe a striated membrane domain that did not at least partially face a PMA mitochondrion (Figure S3). These observations suggest a novel form of contact between the calyx and the type I HC that is mediated, on the HC side, by close association with a mitochondrion. These observations suggest a novel form of contact between the calyx and the type I HC that is mediated, on the HC side, by close association with a mitochondrion. Although we could occasionally resolve electron‐dense material in the synaptic cleft adjacent to the plasma membrane (Figure 1F,G), we could not determine whether these structures were present in every case or were continuous with the striations at the HC and calyx positions. Notably, we did not detect ribbons with synaptic vesicles in close proximity (within 100 nm) to PMA mitochondria.

Using TEM, we observed that PMA mitochondria in type I HCs first appear relatively late during mouse development, around weaning. 10% of type I HCs had at least one PMA mitochondrion at P10‐14 (*n* = 39 HC), with 24% at P18 (*n* = 17), 84% at P25 (*n* = 37), and 85% in adults >P42 (*n* = 12) (Table S1). During development, the calyx is growing and encapsulating the type I HCs from the base to the apex and appears to be fully mature from P14 (Warchol et al. 2019). This suggests that PMA mitochondria appear after full development of the calyx. It is interesting to note that the appearance of large mitochondria in the basal pole of type I HCs has recently been described in adult mice (Michanski et al. 2023).

In adult rodents, ribbons at synapses become sparse in mouse type I HCs (Favre and Sans 1978; Warchol et al. 2019) while floating ribbons, which are not attached to the presynaptic active zone, become predominant (Michanski et al. 2023). Multiple forms of evidence suggest that NQT becomes a dominant form of synaptic activity in type I HCs as mice mature, while both quantal and non‐quantal forms are common in developing animals (reviewed in (Eatock 2025)). The concomitant appearance of striated contacts facing PMA mitochondria with NQT synaptic maturation raises the possibility that these structures play a role in NQT. PMA mitochondria are confined to the region of the type I HC below the nucleus. This basal localization is functionally relevant because NQT depends on ions traversing voltage‐gated channels in both the presynaptic type I membrane and the postsynaptic calyx membrane (Contini et al. 2020; Govindaraju et al. 2023), and these channels are concentrated at the base of the synaptic cleft (Lysakowski et al. 2011). PMA mitochondria could modulate NQT at this site by regulating local ions, such as calcium (Billups and Forsythe, 2002), or by supplying energy to presynaptic ion pumps (Devine and Kittler 2018). Over longer timescales, calyx height—which in simulations affects both the magnitude and speed of NQT (Govindaraju et al. 2023)—may set the overall parameters of NQT, while PMA mitochondria fine‐tune these processes through rapid changes in local ion buffering and ATP supply.

How do PMA mitochondria in type I HCs compare to other mitochondria‐plasma membrane contact sites? Such contacts vary in their distance from the plasma membrane, ranging from 100 nm to as close as 20 nm in gap junction plates (Cetin‐Ferra et al. 2023). The 10–15 nm gap that we observed between PMA mitochondria and the plasma membrane approaches the one at gap junction plates (∼20 nm). Gap junctions, however, have never been described in HCs, including type I HCs (Yamashita and Ohmori 1991). Moreover, the predominant connexins of inner ear sensory epithelia (Cx26 and Cx30) show little or no expression in either rat inner ear HCs (Jagger and Forge 2006) or in mouse vestibular HCs (Lee et al. 2015). Remarkably, only two other examples in the literature show a comparably narrow gap between the mitochondria and the plasma membrane, follicular cells of the bat thyroid (Nunez 1971) and mouse photoreceptors (Meschede et al. 2020), as reviewed in (Casler et al. 2025; Montes de Oca Balderas 2021). In mouse photoreceptors, these contact sites are proposed to regulate Ca2+ fluxes. Interestingly, in those cases, the mitochondria are symmetrically mirrored by apposed mitochondria in adjacent cells, a configuration proposed to be linked to cellular synchronization. In type I HCs, we found that a PMA mitochondrion was mirrored by a mitochondrion in the apposed calyx in 62% of the mitochondria we examined (*n* = 93 PMA mitochondria in 3 segmented cells). However, these calyceal mitochondria were never as closely apposed to the plasma membrane as the PMA of type I HCs (Figure 1B,C,F,H, and Figures S2 and S3).

Another type of striated organelle has occasionally been described in association with HC mitochondria, though they lack bona fide mitochondria‐plasma membrane contact sites. The striated organelles are cytoskeletal structures located at the cuticular plate, a cytoplasmic zone just under the stereocilia bundle, where the organelles are proposed to play a structural role. Striated organelles are spaced approximately 100–150 nm apart, are absent on the basal side of the cell, are not specific to type I HCs, and are found in all cochlear and vestibular HCs (Vranceanu et al. 2012). In contrast, the striations we describe here have a much smaller periodicity of 15 nm. Therefore, the striated organelle is structurally and spatially distinct from the striated contacts reported here.

A distinctive feature of type I HCs is a structure observed via freeze fracture at calyceal contacts, revealing a pattern of intramembrane particle (IMP) distribution in “septate‐like” junctions (Sousa et al. 2009). These HC structures have a periodicity of 15–20 nm, similar in size to the striations we described here. In vertebrates, septate‐like junctions (SJs) have been described exclusively at the paranodal regions of myelinated axons, characterized by their morphological appearance and the presence of vertebrate homologues of *Drosophila* SJ proteins, such as Caspr (the homolog of *Drosophila* neurexin IV). Caspr is critical for targeting paranodal proteins and segregating membrane domains, functioning as a barrier to separate Na^+^ and K^+^ channels (Faivre‐Sarrailh 2020). Notably, Caspr has been localized to the calyceal membrane at the junctional contact with type I HCs. Using Caspr knock‐out mice, it was demonstrated that Caspr is essential for maintaining functional junctional contact between the calyx and type I HCs (Sousa et al. 2009). Future studies could explore whether the striated contacts associated with PMA mitochondria are enriched with Caspr and assess their functional impact.

Beyond these local junctional specializations, the striated contacts may also share organizational principles with transsynaptic structures described in conventional chemical synapses. Conventional chemical synapses are organized into presynaptic, cleft, and postsynaptic compartments, and it has shown these are structurally coupled across the cleft. Super‐resolution microscopy revealed alignment of presynaptic vesicle‐release machinery and postsynaptic receptor–scaffolding complexes into transsynaptic nanocolumns (Tang et al. 2016). More recently, electron tomography showed that proteinaceous columns traverse the cleft, linking presynaptic vesicles to postsynaptic density scaffolding via transmembrane structures; these transsynaptic assemblies physically bridge all three compartments (Cole and Reese 2023). Striated contacts in the mouse vestibular sensory epithelium could be analogous to these assemblies, potentially representing specialized functional domains that align pre‐ and postsynaptic elements across the hair cell–calyx interface. Further research is needed to uncover the structural and molecular components of these novel striated contacts and the molecular mechanisms underlying mitochondria‐plasma membrane tethering in type I HCs. Understanding their role will provide valuable insights into synaptic transmission and broader cellular communication processes.

## 2 Materials and Methods

### 2.1 Animals

All mice were C57BL/6J background. Both genders were used in all studies. The University of Washington School of Medicine (Seattle, WA) conducted all animal procedures using approved protocols from their animal care and use committee that conform to the standards of the American Veterinary Medical Association and the National Institutes of Health.

### 2.2 Electron Microscopy—Serial Block Face SEM Sample Preparation

Utricles were fixed in 4% glutaraldehyde in 0.1 M sodium cacodylate buffer then post‐fixed in osmium ferrocyanide (2% osmium, 1.5% potassium ferrocyanide, in 0.1 M cacodylate buffer). Then utricles were incubated in 1% thiocarbohydrazide for 20 min, then in 2% osmium tetroxide for 30 min, then in 1% uranyl acetate overnight in the refrigerator. Next, utricles were stained using Walton's lead aspartate for 30 min at 60°C, then dehydrated in ice cold 30%, 50%, 70%, and 95% ethanol, then 2 changes of 100% ethanol and two changes of propylene oxide. Rinses were performed at each step, and every step was performed at room temperature unless noted. Utricles were embedded in Durcupan resin. The block was attached to the mounting pin using conductive epoxy made by Circuitworks and supplied by SPI. The block was trimmed to ∼ 750 µm on each side and coated with gold using a sputter coater.

### 2.3 Electron Microscopy—Serial Block Face SEM Acquisition and Segmentation

Image stack acquisition was performed on a Teneo VS (ThermoFisher Scientific) in LoVac (30 Pa of water vapor) in ThruSight mode with an acquisition voxel size of 10 × 10 × 20 nm and sectioning every 40 nm. The data set was then binned twice in x,y to end up with isotropic voxels of 20 × 20 × 20 nm.

The extracellular environment was outlined using the ilastik carving mode (Berg et al., 2019) to generate a first dataset in order to later mask out all the mitochondria that don't belong to the Type I HC. All mitochondria of the original dataset were segmented using the pixel classification mode of Ilastik using seeds in the Type I HC mitochondria. In parallel, the same original data set was treated with the pretrained CebraNet model to reveal all cellular membranes. Finally combining the three resulting datasets, we could isolate the Type I HC mitochondria and run their morphological analysis in Amira. The nucleus was segmented using the ilastik carving mode. Beyond classical 3D morphometrics (including area and Feret diameters), we measured the proportion of contact between every Type I HC mitochondrion and the plasma membrane by dividing half of the Area of the interaction plate between the mitochondria and the plasma membrane by the Area of the same mitochondria. Movies were generated in Amira and the color coding of each mitochondrion corresponds to its proportion of contact with the Type I HC plasma membrane which can span from 0% (no contact, red) to about 50% (fully apposed mitochondria, white).

To extract the contact distance of the PMA to the plasma membrane, in each segmented SBF cell, we created a Euclidean distance map from the segmented cell body of the type I HC. For every segmented mitochondria, the minimum value was extracted (Amira), corresponding to the minimal distance between the mitochondria and the plasma membrane at one point of the mitochondria.

### 2.4 Electron Microscopy—Sample Preparation

Utricles and posterior crista ampullae were prepared as described in (Warchol et al. 2019). Briefly, temporal bones were isolated and fixed in 2.5% glutaraldehyde in 0.1 M cacodylate buffer, rinsed and post‐fixed with 2% osmium tetroxide in cacodylate buffer. The end organs were dissected and embedded in Eponate (Ted Pella Inc. #18010).

### 2.5 Electron Microscopy—Serial Tomography Acquisition and Segmentation

350 nm semithin sections were performed using a Leica UC7 ultramicrotome and post‐stained with 2% uranyl acetate (16 min) and Reynolds’ lead citrate (8 min). Images were taken with a Tecnai G2 microscope (FEI) at 200 kV. For tomography acquisitions, tilted images (+60°/–60° according to a Saxton scheme) were acquired using Xplorer 3D (FEI) with a Veleta camera (Olympus, Japan). Tilted series alignment and tomography reconstruction were performed using IMOD (Mastronarde and Held 2017). The segmentation and movie generation were carried out in Amira. To segment the uneven curvature of the plasma membrane, we regularly placed landmarks to generate a surface that was dilated and used as a mask to reveal its striation in an *en face* view.

## Conflicts of Interest

The authors declare no conflicts of interest.

## Supporting information




**Supporting File 1**: boc70082‐sup‐0001‐SuppMat.docx.

**Supporting File 2**: boc70082‐sup‐0002‐Video1.avi.

**Supporting File 3**: boc70082‐sup‐0003‐Video2.mp4.

## Data Availability

The data that support the findings of this study are available from the corresponding author upon request.

## References

[boc70082-bib-0001] Berg, S. , D. Kutra , T. Kroeger , et al. 2019. “ilastik: Interactive Machine Learning for (bio)Image Analysis.” Nature Methods 16: 1226–1232. 10.1038/s41592-019-0582-9 31570887

[boc70082-bib-0030] Billups, B. , and I. D. Forsythe . 2002. “Presynaptic Mitochondrial Calcium Sequestration Influences Transmission at Mammalian Central Synapses.” The Journal of Neuroscience 22, no. 14: 5840–5847. 10.1523/jneurosci.22-14-05840.2002. PMC6757942 12122046

[boc70082-bib-0002] Casler, J. C. , M. V. Neto , T. Burgoyne , and L. L. Lackner . 2025. “Mitochondria‐Plasma Membrane Contact Sites: Emerging Regulators of Mitochondrial Form and Function.” Contact (Thousand Oaks) 8: 25152564251332141. 10.1177/25152564251332141 PMC12033498 40291948

[boc70082-bib-0003] Cetin‐Ferra, S. , S. C. Francis , A. T. Cooper , et al. 2023. “Mitochondrial Connexins and Mitochondrial Contact Sites with Gap Junction Structure.” International Journal of Molecular Sciences 24: 9036. 10.3390/ijms24109036 PMC10219162 37240383

[boc70082-bib-0004] Cole, A. A. , and T. S. Reese . 2023. “Transsynaptic Assemblies Link Domains of Presynaptic and Postsynaptic Intracellular Structures Across the Synaptic Cleft.” Journal of Neuroscience 43: 5883–5892. 10.1523/JNEUROSCI.2195-22.2023 PMC10436760 37369583

[boc70082-bib-0005] Contini, D. , G. R. Holstein , and J. J. Art . 2020. “Synaptic Cleft Microenvironment Influences Potassium Permeation and Synaptic Transmission in Hair Cells Surrounded by Calyx Afferents in the Turtle.” Journal of Physiology 598: 853–889. 10.1113/JP278680 PMC7024053 31623011

[boc70082-bib-0006] Devine, M. J. , and J. T. Kittler . 2018. “Mitochondria at the Neuronal Presynapse in Health and Disease.” Nature Reviews Neuroscience 19: 63–80. 10.1038/nrn.2017.170 29348666

[boc70082-bib-0007] Eatock, R. A. 2025. “Functional Development and Differentiation of Mammalian Vestibular Hair Cells and Their Synapses.” Current Topics in Developmental Biology 165: 235–306. 10.1016/bs.ctdb.2025.07.001 40973231

[boc70082-bib-0029] Sadeghi, S. G. , S. J. Pyott , Z. Yu , and E. Glowatzki . 2014. “Glutamatergic Signaling at the Vestibular Hair Cell Calyx Synapse.” The Journal of Neuroscience 34, no. 44: 14536–14550. 10.1523/jneurosci.0369-13.2014. PMC4212060 25355208

[boc70082-bib-0008] Eatock, R. A. , and J. E. Songer . 2011. “Vestibular Hair Cells and Afferents: Two Channels for Head Motion Signals.” Annual Review of Neuroscience 34: 501–534. 10.1146/annurev-neuro-061010-113710 21469959

[boc70082-bib-0009] Faivre‐Sarrailh, C. 2020. “Molecular Organization and Function of Vertebrate Septate‐Like Junctions.” Biochimica et Biophysica Acta (BBA)—Biomembranes 1862: 183211. 10.1016/j.bbamem.2020.183211 32032590

[boc70082-bib-0010] Favre, D. , and A. Sans . 1978. “The Development of Vestibular Efferent Nerve Endings During Cat Maturation: Ultrastructural Study.” Brain Research 142: 333–337. 10.1016/0006-8993(78)90639-x 630388

[boc70082-bib-0011] Govindaraju, A. C. , I. H. Quraishi , A. Lysakowski , R. A. Eatock , and R. M. Raphael . 2023. “Nonquantal Transmission at the Vestibular Hair Cell‐Calyx Synapse: KLV Currents Modulate Fast Electrical and Slow K+ Potentials.” PNAS 120: e2207466120. 10.1073/pnas.2207466120 PMC9926171 36595693

[boc70082-bib-0012] Jagger, D. J. , and A. Forge . 2006. “Compartmentalized and Signal‐Selective Gap Junctional Coupling in the Hearing Cochlea.” Journal of Neuroscience 26: 1260–1268. 10.1523/JNEUROSCI.4278-05.2006 PMC6674557 16436613

[boc70082-bib-0013] Lee, M. Y. , T. Takada , Y. Takada , et al. 2015. “Mice with Conditional Deletion of Cx26 Exhibit no Vestibular Phenotype Despite Secondary Loss of Cx30 in the Vestibular End Organs.” Hearing Research 328: 102–112. 10.1016/j.heares.2015.07.018 PMC4581988 26232528

[boc70082-bib-0014] Lysakowski, A. 1999. “Development of Synaptic Innervation in the Rodent Utricle.” Annals of the New York Academy of Sciences 871: 422–425. 10.1111/j.1749-6632.1999.tb09209.x PMC3809015 10372096

[boc70082-bib-0015] Lysakowski, A. , S. Gaboyard‐Niay , I. Calin‐Jageman , S. Chatlani , S. D. Price , and R. A. Eatock . 2011. “Molecular Microdomains in a Sensory Terminal, the Vestibular Calyx Ending.” Journal of Neuroscience 31: 10101–10114. 10.1523/JNEUROSCI.0521-11.2011 PMC3276652 21734302

[boc70082-bib-0016] Lysakowski, A. , A. C. Govindaraju , and R. M. Raphael . 2022. “Structural and Functional Diversity of Mitochondria in Vestibular/Cochlear Hair Cells and Vestibular Calyx Afferents.” Hearing Research 426: 108612. 10.1016/j.heares.2022.108612 PMC12058273 36223702

[boc70082-bib-0017] Mastronarde, D. N. , and S. R. Held . 2017. “Automated Tilt Series Alignment and Tomographic Reconstruction in IMOD.” Journal of Structural Biology 197: 102–113. 10.1016/j.jsb.2016.07.011 PMC5247408 27444392

[boc70082-bib-0018] Meschede, I. P. , N. C. Ovenden , M. C. Seabra , et al. 2020. “Symmetric Arrangement of Mitochondria: Plasma Membrane Contacts between Adjacent Photoreceptor Cells Regulated by Opa1.” Proceedings of the National Academy of Sciences 117: 15684–15693. 10.1073/pnas.2000304117 PMC7355040 32571921

[boc70082-bib-0019] Michanski, S. , T. Henneck , M. Mukhopadhyay , et al. 2023. “Age‐Dependent Structural Reorganization of Utricular Ribbon Synapses.” Frontiers in Cell and Developmental Biology 11: 1178992. 10.3389/fcell.2023.1178992 PMC10447907 37635868

[boc70082-bib-0020] Montes de Oca Balderas, P. 2021. “Mitochondria‐Plasma Membrane Interactions and Communication.” Journal of Biological Chemistry 297: 101164. 10.1016/j.jbc.2021.101164 PMC8503596 34481840

[boc70082-bib-0021] Nunez, E. A. 1971. “Secretory Processes in Follicular Cells of the Bat Thyroid. II. The Occurrence of Organelle‐Associated Intercellular Junctions During Late Hibernation.” American Journal of Anatomy 131: 227–239. 10.1002/aja.1001310207 5575891

[boc70082-bib-0022] Songer, J. E. , and R. A. Eatock . 2013. “Tuning and Timing in Mammalian Type I Hair Cells and Calyceal Synapses.” Journal of Neuroscience 33: 3706–3724. 10.1523/JNEUROSCI.4067-12.2013 PMC3857958 23426697

[boc70082-bib-0023] Sousa, A. D. , L. R. Andrade , F. T. Salles , et al. 2009. “The Septate Junction Protein Caspr Is Required for Structural Support and Retention of KCNQ4 at Calyceal Synapses of Vestibular Hair Cells.” Journal of Neuroscience 29: 3103–3108. 10.1523/JNEUROSCI.4868-08.2009 PMC2668863 19279247

[boc70082-bib-0024] Spaiardi, P. , W. Marcotti , S. Masetto , and S. L. Johnson . 2022. “Signal Transmission in Mature Mammalian Vestibular Hair Cells.” Frontiers in Cellular Neuroscience 16: 806913. 10.3389/fncel.2022.806913 PMC9353129 35936492

[boc70082-bib-0025] Tang, A.‐H. , H. Chen , T. P. Li , S. R. Metzbower , H. D. MacGillavry , and T. A. Blanpied . 2016. “A Trans‐Synaptic Nanocolumn Aligns Neurotransmitter Release to Receptors.” Nature 536: 210–214. 10.1038/nature19058 PMC5002394 27462810

[boc70082-bib-0026] Vranceanu, F. , G. A. Perkins , M. Terada , R. L. Chidavaenzi , M. H. Ellisman , and A. Lysakowski . 2012. “Striated Organelle, a Cytoskeletal Structure Positioned to Modulate Hair‐Cell Transduction.” Proceedings of the National Academy of Sciences 109: 4473–4478. 10.1073/pnas.1101003109 PMC3311375 22396594

[boc70082-bib-0027] Warchol, M. E. , R. Massoodnia , R. Pujol , B. C. Cox , and J. S. Stone . 2019. “Development of Hair Cell Phenotype and Calyx Nerve Terminals in the Neonatal Mouse Utricle.” Journal of Comparative Neurology 527: 1913–1928. 10.1002/cne.24658 PMC7362884 30724338

[boc70082-bib-0028] Yamashita, M. , and H. Ohmori . 1991. “Synaptic Bodies and Vesicles in the Calix Type Synapse of Chicken Semicircular Canal Ampullae.” Neuroscience Letters 129: 43–46. 10.1016/0304-3940(91)90716-7 1922969

