# Supplementary material for: A Novel Striated Membrane Contact Site in Vestibular Hair Cells
Source: Biol Cell. 2026 Sep 29;118(10):e70082. doi: 10.1111/boc.70082 (PMC13621331; doi:10.1111/boc.70082)
Supplement: Supplementary file 1 [file BOC-118-e70082-s001.docx]

***Supplementary Information***

**Supplementary Figure 1,** PMA mitochondria were observed in different zones of the utricle - the striola and extrastriola. PMA mitochondria were also seen in the posterior crista ampullaris.

A: TEM overview of the whole utricle. The area outlined in magenta corresponds to the striola, identified by the numerous multicalyces. The '+' symbols in yellow represent type I cells, identified according to the presence (type I) of a calyx with at least one PMA mitochondrion. Scale bar, 50 µm. B-D: Examples of PMA mitochondria in the utricle’s extrastriola (B) and striola (C), and the posterior crista ampullaris (D), scale bar, 1 µm. The posterior crista ampullaris image was obtained following a different protocol (SBF), explaining the different contrast.

**Supplementary Figure 2,** Examples of mirroring mitochondria in the type I HC and neural calyx

Transverse TEM image of the basal side of a type I HC. Mitochondria in the calyx are highlighted with green * while PMA in the type I HC are in yellow *. Scale bar 500 nm in both the main image and its insert.

**Supplementary Figure 3 -** Examples of striated contact revealed in different electron tomographs

t.I pm : plasma membrane on the Type I HC; C pm : plasma membrane of the Calyx ; C : Calyx ; Yellow asterisk : PMA mitochondria ; Green asterisk : Calyx mitochondria ; sc : synaptic cleft.

**Supplementary Figure 4 -** Characteristics of the mitochondria in type I HC from Serial BlockFace imaging

A and B: Bar graphs presenting the distribution of the distance of each mitochondrion to the plasma membrane (A) and the distribution of the proportion of contact of each mitochondrion with the plasma membrane (B). The bar graph in B suggests a bimodal distribution of the mitochondria in Type I HC. C: Scatter plot presenting the proportion of contact of each mitochondrion with the plasma membrane (y axis) versus the overall surface of the mitochondria (x axis). The mitochondria showing the higher value of surface are PMA mitochondria (with a high value of proportion of contact with the PM). The analyzed cells portions did not include the cuticular plate, hence the presented figures only correspond to the mitochondria population that is found on the basal main cell body of the Type I HC (n=325 mitochondria from 3 different cells imaged by Serial BlockFace SEM).

A

B

C

**Supplementary Table 1 -** PMA mitochondria become dominant at P25 in type I HC

|  | *HCs observed* | *HCs with apposed mitochondria** | *Frequency* |
| --- | --- | --- | --- |
| P10-P14 | **39** | **4** | 10% |
| P18 | **24** | **6** | 25% |
| P25 | **37** | **31** | 84% |
| Young adult | **12** | **10** | 85% |

*The number of type I HCs with at least one PMA mitochondrion was reported over the total number of type I HCs observed using TEM at different developmental stages in the mouse.

**Supplementary Video 1:** SBF of a type I HC revealing (PMA) mitochondria in yellow and non-apposed mitochondria in red, nucleus in blue.

**Supplementary Video 2:** Serial tomography of a striated junction with a PMA in yellow, the plasma membrane of the Type I HC in grey, and plasma membrane of the calyx in green. The volume of the PMA is transformed into discontinuous very small orange dots to better see the striations on both plasma membranes; two serial tomographs are joined, hence the space in between them.
